# Reliability of Isokinetic Strength Assessments of Knee and Hip Using the Biodex System 4 Dynamometer and Associations With Functional Strength in Healthy Children

**DOI:** 10.3389/fspor.2022.817216

**Published:** 2022-02-24

**Authors:** Vanessa van Tittelboom, Ipek Alemdaroglu-Gürbüz, Britta Hanssen, Lieve Heyrman, Hilde Feys, Kaat Desloovere, Patrick Calders, Christine Van den Broeck

**Affiliations:** ^1^Department of Rehabilitation Sciences, Ghent University, Ghent, Belgium; ^2^Department of Rehabilitation Sciences, Katholieke Universiteit Leuven, Leuven, Belgium; ^3^Department of Physiotherapy and Rehabilitation, Hacettepe University, Ankara, Turkey

**Keywords:** isokinetic dynamometry, lower limbs, children, reliability, functional strength

## Abstract

**Background:**

This study aimed to analyze the reliability of concentric isokinetic strength assessments (knee and hip) using the Biodex System 4 in healthy children and assess the association with functional strength tests (sit-to-stand [STS], lateral-step-up [LSU]).

**Methods:**

19 children (6–12 years) were included. Knee and hip flexion and extension, and hip abduction and adduction were tested at 60 and 90°/s.

**Results:**

Relative and absolute reliability at 60°/s tended to show better results compared to those at 90°/s. Intra class correlations (ICCs) of knee flexion and extension at 60°/s were good (0.79–0.89). For hip flexion, extension, abduction and adduction at 60°/s ICCs were moderate to good (0.53–0.83). The smallest detectable change (SDC) values (expressed in %) were highly variable. The SDC% for knee flexion and extension and hip abduction at 60°/s were around 50%. Positive associations were found between hip extension and abduction isokinetic strength and the STS test.

**Conclusion:**

Concentric isokinetic strength assessments in healthy children using the Biodex System 4 were found reliable for knee flexion and extension and hip abduction. Limited associations were found between concentric isokinetic strength tests and functional strength tests.

## Introduction

Muscle strength is crucial for many daily life activities such as walking or jumping (De Ste Croix et al., 2003; van der Krogt et al., 2012; Santos et al., 2013; Eliakim et al., 2019). Youngsters with inadequate muscular strength are less likely to gain competence and confidence in their motor skill abilities and will have limited participation in exercise, games, and sports activities (Faigenbaum et al., 2013). Caregivers should therefore recognize muscle strength as a prerequisite of global health (Faigenbaum et al., 2013; Eliakim et al., 2019).

Resistance training has shown to be safe, to improve muscular fitness and to reduce the risk of injury in children and adolescents (Faigenbaum et al., 2013). To analyze possible effects of resistance training, a valid and reliable tool for muscle strength assessment is indispensable. Currently, several options are available: manual tests, handheld dynamometry, the 1-repetition maximum test, functional strength tests and isokinetic dynamometry (Schwartz et al., 1992; van den Beld et al., 2006; Santos et al., 2013; Aertssen et al., 2016). Isokinetic dynamometry is considered the gold standard for objectifying strength (Ayalon et al., 2000; Wiggin et al., 2006; Tsiros et al., 2011).

In many functional activities, the knee extensors and flexors play an important role by stabilizing the knee joint (Mikesky et al., 2000; Fagher et al., 2016; Munoz-Bermejo et al., 2019). A recent review, including 10 studies, on the reliability of isokinetic strength measurements of the knee in children (healthy and with cerebral palsy) revealed poor to excellent (intra-class correlation [ICC], 0.31–0.99) levels of relative reliability for concentric movements (Munoz-Bermejo et al., 2019). Nevertheless, the heterogeneity of the included studies (e.g., isokinetic devices, populations, protocols) hamper general conclusions.

Weakness of the hip abductors and hip flexors affect a normal gait pattern by increasing total muscle cost (van der Krogt et al., 2012). Hip flexors are important during swing phase, and the gluteus medius is crucial for vertical support (Liu et al., 2008; Hall et al., 2011). To our knowledge, only two studies focused on the evaluation of isokinetic strength assessment of the hip joint in children (Molnar et al., 1979; Burnett et al., 1990). Burnett et al. (1990) reported poor to good relative reliability for concentric isokinetic measurements of the hip flexors, extensors, abductors and adductors with ICCs ranging from 0.49 to 0.75. Molnar and Alexander concluded, based on score deviations, that the isokinetic technique is reliable for muscle strength assessment of the hip flexors, extensors and abductors in children (Molnar et al., 1979).

Absolute reliability provides clinical guidance for assessing real changes (Dvir, 2003). Only few studies focusing on isokinetic assessment of the knee in children reported absolute reliability results. They found results for the standard error of measurement (SEM%) results ranging from 5.2 to 13.9% and for the smallest detectable change (SDC%) ranging from 14.4 to 38.5% (Munoz-Bermejo et al., 2019). However, no conclusions can be drawn due to several discrepancies between these studies. No data are available for the hip.

Pediatric physical therapy interventions ultimately aim to target activity and participation levels. Functional strength tests are often used to evaluate functional capacity in children. Overall, a low to moderate association (*r* = 0.42–0.69) has been previously shown between isometric strength measurements and functional strength tests of the lower limbs (Aertssen et al., 2016).

The main aim of the present study is to analyze the relative and absolute reliability of concentric isokinetic strength assessments of the knee and the hip using the Biodex System 4 in healthy children. Additionally, the possible relationship between isokinetic dynamometry of several relevant muscle groups and functional strength tests were explored.

## Methods

### Study Design

To investigate intra-tester reliability of the concentric isokinetic strength assessments of the knee and hip, children were tested twice by the same pediatric physical therapists and on the same time of the day with a minimum interval of 1 week and a maximum interval of 2 weeks in between testing. During the first session, children also performed functional strength tests after the isokinetic strength assessments to enable analysis of the possible relationships between functional strength and isokinetic strength.

The clinical trial registration date and number of this study are, respectively, 18/07/2019 and NCT04024592.

### Participants

Healthy children were recruited from several primary schools in Flanders, Belgium and via acquaintances of the authors between February and March 2018. Children with chronic orthopedic, neurological, or cardiorespiratory problems were excluded. All parents and 12-year-old children signed an informed consent form. In this document they confirmed that they received sufficient oral and written information regarding the study. For all participants an adapted information form was given with easy to understand explanation of the testing protocol. This study was approved by the institutional ethics committee of Ghent University Hospital (EC/2017/1674).

### Testing Protocol

All children were tested separately. Participants were asked to create the same conditions for both test occasions (e.g. physical activity and sleep) and to limit physical activity to their habitual activities before testing. Physical activity of the last 24 hours was questioned by means of a standardized interview. Before each session, a warm-up period of 2 min on a cycle ergometer at a low pre-set intensity of 25 Watt was performed, similar to warming ups provided in previous research (Wiggin et al., 2006; Fagher et al., 2016). Leg dominance was determined by asking the child to kick a ball three times.

**Figure 1:**
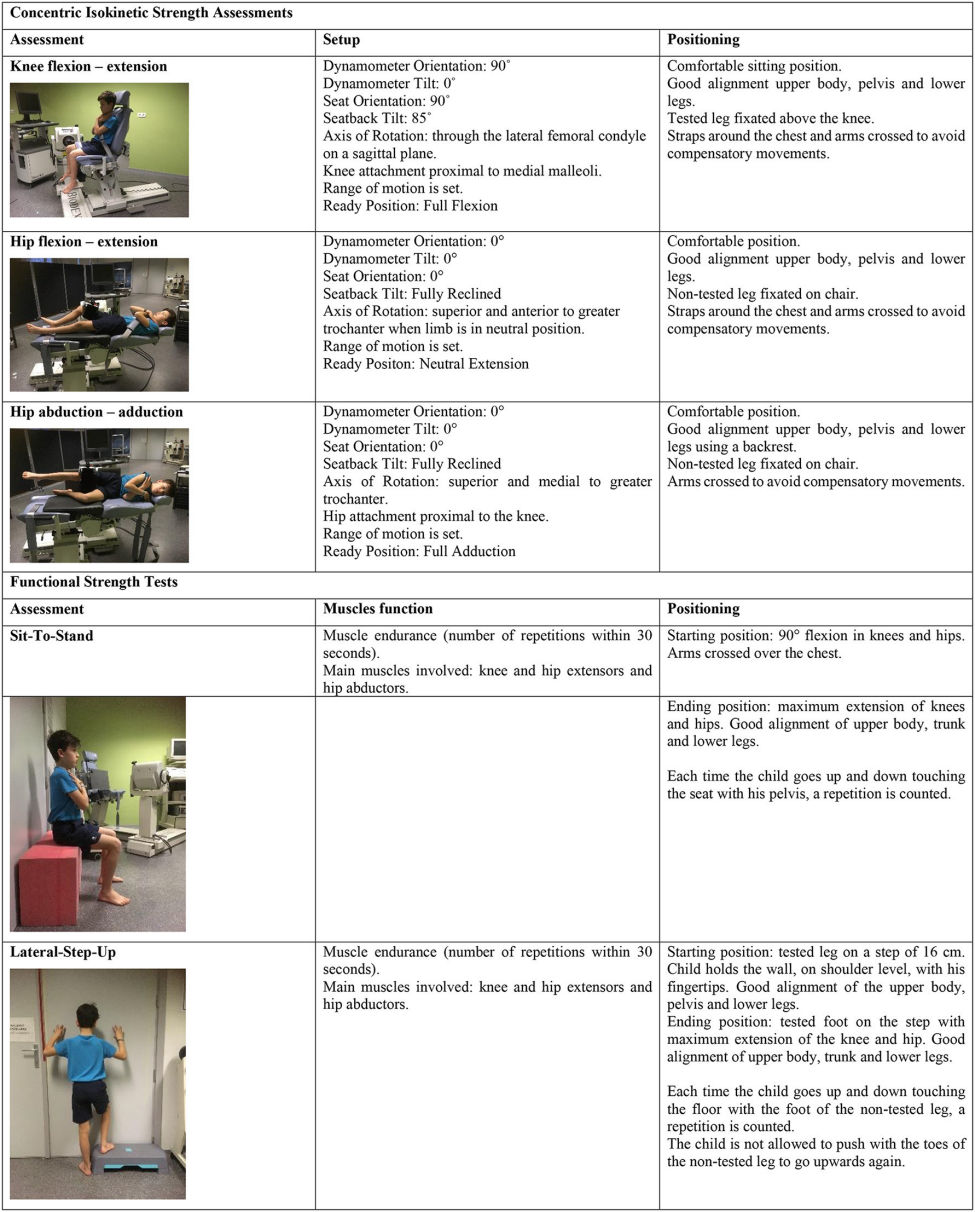
Description of concentric isokinetic strength assessments and functional strength tests.

A detailed description of the testing procedures of the concentric isokinetic strength assessments and functional strength tests is presented in Figure 1.

#### Concentric Isokinetic Strength Assessment

All concentric isokinetic measurements were performed using a Biodex System 4 (Model 850-230, Universal Pro Single Chair Assy, Biodex Medical Systems, Inc., Shirley, New York, USA). The measurements included knee flexion and extension, and hip flexion, extension, abduction, and adduction. Assessment protocols of the manufacturer were applied and dynamometer setup specifications during the first session (e.g., seat pan position, dynamometer height and leg cuff position) were noted and repeated during the second session. For hip abduction and adduction, a standardized test setup based on the study of Meyer et al. (2013) was used. Participants laid in side-lying, using a backrest to provide stability and to avoid compensatory movements. In the current study, no pediatric attachments were used (Fagher et al., 2016). The leg length of the included children was sufficiently long to allow standardized positioning, both for knee and hip assessments. Moreover, the pediatric hip attachment does only enable measurements in a standing position.

The dominant leg was always tested first and both legs were tested at a rate of 60° and 90° per second (/s), as these velocities seem to represent velocities used during activities of daily life (Li et al., 1996; Ayalon et al., 2000; Wiggin et al., 2006). Moreover, an angular velocity of 60°/s has been used in previous research (Ayalon et al., 2000; Wiggin et al., 2006; Santos et al., 2013) whereas 90°/s has not yet been explored in children. Lower (e.g. 30°/s) and higher (e.g. 180°/s) velocities were not selected since children seem to get discouraged or show difficulties in consistently generating torques at these velocities, respectively (Wiggin et al., 2006; Santos et al., 2013). Before each test, children were asked to perform three submaximal and one maximal repetition to become familiar with the test procedure. During the test procedure, children were asked to perform three consecutive maximal repetitions for each tested muscle group. There was no pause in between the repetitions of the practice session, neither in between the repetitions of the test session. The time between the practice moment and the actual test was 30 s. Between the two velocity conditions, children could rest for 1 min. Between each tested muscle group, participants were again allowed to rest for 2 min. The assessors gave standardized verbal feedback, encouraging the children to perform at maximal strength and to move through the total preset range of motion. Also, constant update regarding the remaining repetitions was provided. For each test, the values of three consecutive isokinetic contractions were used for statistical analyses. To ensure that best effort was obtained only assessments for which the coefficient of variance was ≤ 20% were included (Wiggin et al., 2006). Limb weight correction was not applied. The use of gravity correction in children is unclear. Literature suggests that in adult error levels in isokinetic measurements occur when gravity is uncorrected. Nevertheless, according to Jones and Stratton (2000), correction using adult procedures is thought to overestimate gravitational torque in children, as these do not account for the elastic components of the growing muscle-joint system. From our observations, it is very difficult for children to relax and obtain a correct limb weight. This is even more so when total limb weight has to be considered while measuring hip strength (Burnett et al., 1990). Furthermore, gravity correction was not essential to the aim of our study, which was to analyze the reliability of isokinetic strength assessments. The weight of the limb was assumed to be the same within the 2-week interval period, and the testing positions were standardized.

Peak torque (PT; expressed in Newton meter, Nm) and mean PT (MPT; Nm) were identified for each assessment. Peak torque was defined as the highest force output at any moment during a repetition. MPT was the average of the peak torque values obtained during a series of three repetitions. The MPT may be considered a better estimate of overall function than PT given that function is dependent on repetition of movement. PT and MPT values have been found to be reliable measures of muscle performance and are often used in previous research articles covering isokinetic strength assessments in children. Using those values, the data of the current study could be compared with those of previously published studies.

#### Functional Strength Tests

Participants performed the sit-to-stand (STS) test and lateral step-up (LSU) test. Those functional tests are frequently used for assessment of functional strength in children, both in research and clinical practice. Test-retest reliability of these tests range from 0.70 to 0.79 in children aged from seven to 10 years (y) old (Aertssen et al., 2016).

Participants had three practice trials to ensure good understanding of the test. For all functional strength tests, the number of correct trials within 30 s were counted. Children were motivated verbally by the assessors. A 2-min rest period was implemented between the different functional tests. There was a 15-min rest period between the isokinetic strength assessments and the functional strength tests.

Only the most related muscles to each functional strength test were used for analysis.

### Statistical Analysis

#### Descriptive Statistics

Agreement between both test occasions and potential differences between the dominant and the non-dominant side and between the two velocities (60°/s and 90°/s) were assessed with a paired t-test.

#### Intra-Tester Reliability

The relative reliability was assessed by means of the 2-way ICC_2.1_ for agreement to determine the test–retest reliability of the isokinetic strength assessments. The ICC values were interpreted based on the classification of Portney and Watkins (2009): <0.5 = poor reliability, 0.5–0.75 = moderate (poor to moderate) reliability, 0.75–0.9 = good reliability, and > 0.90 = excellent reliability. For ICC, a 95% confidence interval was calculated.

Absolute reliability was assessed with the SEM. The SEM agreement was defined by the formula: ($\surd {\text{σ}}_{1-2}^{2}+{\text{σ}}_{\text{residual}}^{2})$ (de Vet et al., 2006). The SEM% was defined as: SEM/mean of all measurements from both test sessions × 100. The SDC is the minimum difference to be considered clinically important and was determined using following formula: 1.96 × √2 × SEM_agreement_. The SDC was also expressed as a percentage value (Copay et al., 2007; Weir, 2009).

#### Associations Between Functional Strength Tests and Concentric Isokinetic Dynamometry

Associations were determined by calculating the Pearson correlation coefficients (*r*-value), relating the outcomes of the isokinetic strength assessments of the first test occasion with the functional strength tests scores. The interpretation of the *r*-value was done based on following distribution: *r* < 0.3: very weak, *r* = 0.3–0.5: weak, *r* = 0.5–0.7: moderate, *r* = 0.7–0.85: strong, *r* = 0.85–0.95: very strong, and *r* > 0.95: excellent correlation (Van Maele et al., 2014).

All statistical analysis were performed with IBM SPSS Version 23.0 (SPSS Inc., Chicago, IL).

## Results

### Participants

A total of 19 healthy children (8 girls, 11 boys) aged 6–12 y (mean 10.0, standard deviation [SD] 1.6) were included in this study. Height of the children ranged from 132 to 161 cm (mean 146, SD 10.1) and weight from 26.8 to 45.9 kg (mean 35.1, SD 7.2).

### Torques

**Figure 2:**
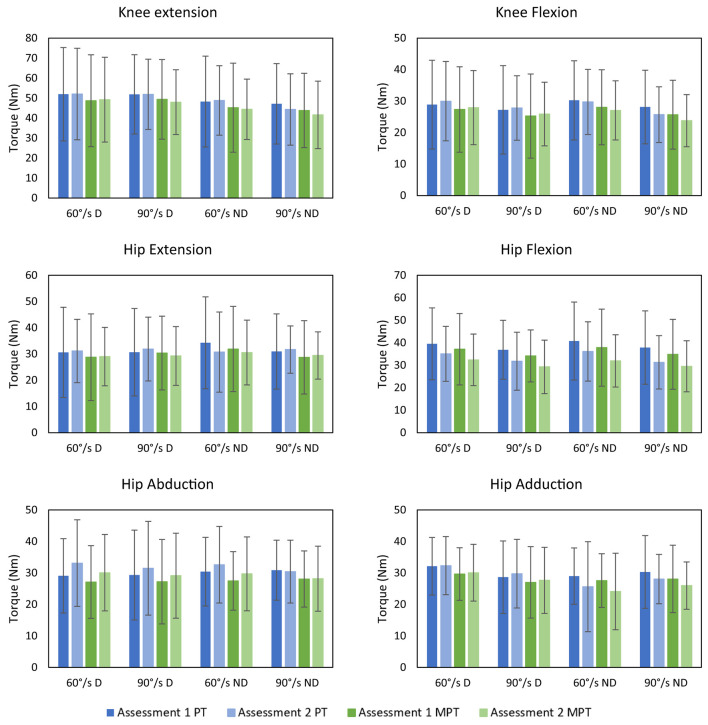
Depiction of the means and standard deviations of the repeated assessments for each joint movement, velocity and dominant and non-dominant leg. D: dominant; MPT: Mean Peak Torque; ND: Non-dominant; Nm: Newton meter; PT: Peak Torque; /s: per second.

The tables of the mean and standard deviation of PT and mean MPT for each assessment can be found in the Appendix Tables A1–A3. Graphical representation of the torques can be found in Figure 2.

There were no significant differences between both test occasions. The torques of the dominant side and the non-dominant side did not show significant differences. Torques performed at 60°/s and 90°/s did not differ significantly from each other, except for hip extension PT and MPT, hip flexion MPT and knee flexion PT of the non-dominant side.

### Intra-Tester Reliability

Reliability results are reported in Tables 1–4. Relative reliability of PT and MPT assessments for knee flexion, knee extension, and hip abduction were good. Moderate relative reliability was found for PT and MPT of hip extension, hip flexion, and hip adduction. Overall, relative reliability at 60°/s tended to show slightly better results than at 90°/s. Absolute reliability, expressed in SEM% and SDC%, tended to show the same pattern as the relative reliability.

**Table 1:** Relative reliability (ICC) of the concentric isokinetic strength assessments at 60°/s.

	**Peak torque**				**Mean peak torque**			
	**Dominant**		**Non-dominant**		**Dominant**		**Non-dominant**	
	**ICC**	**CI (95%)**	**ICC**	**CI (95%)**	**ICC**	**CI (95%)**	**ICC**	**CI (95%)**
Knee extension	**0.87**	0.64–0.96	**0.81**	0.51–0.97	**0.88**	0.65–0.96	**0.79**	0.47–0.93
Knee flexion	**0.84**	0.50–0.95	**0.89**	0.69–0.96	**0.85**	0.53–0.96	**0.88**	0.67–0.96
Hip extension	0.56	0.10–0.81	**0.75**	0.43–0.90	0.53	0.07–0.80	**0.76**	0.44–0.91
Hip flexion	0.62	0.18–0.86	0.69	0.36–0.87	0.71	0.33–0.90	0.73	0.35–0.89
Hip abduction	**0.81**	0.50–0.93	**0.77**	0.45–0.91	**0.83**	0.57–0.94	0.71	0.34–0.89
Hip adduction	**0.75**	0.37–0.91	0.60	0.14–0.85	0.73	0.34–0.91	0.66	0.25–0.93

*Reliability indices classified as good (ICC, 0.75 – 0.90) are shown in bold type. /s, per second; CI, confidence interval; ICC, intra-class correlation coefficient*.

**Table 2:** Relative reliability (ICC) of the concentric isokinetic strength assessments at 90°/s.

	**Peak torque**				**Mean peak torque**			
	**Dominant**		**Non-dominant**		**Dominant**		**Non-dominant**	
	**ICC**	**CI (95%)**	**ICC**	**CI (95%)**	**ICC**	**CI (95%)**	**ICC**	**CI (95%)**
Knee extension	0.59	0.11–0.84	**0.83**	0.61–0.93	0.58	0.11–0.84	**0.80**	0.55–0.92
Knee flexion	**0.75**	0.39–0.92	0.67	0.26–0.88	**0.80**	0.53–0.92	0.62	0.17–0.86
Hip extension	0.52	0.03–0.80	0.67	0.22–0.88	0.50	0.01–0.79	0.64	0.18–0.87
Hip flexion	0.55	0.11–0.82	0.69	0.27–0.87	0.50	0.04–0.79	0.69	0.33–0.87
Hip abduction	**0.83**	0.56–0.94	**0.88**	0.66–0.96	**0.85**	0.61–0.95	**0.88**	0.68–0.96
Hip adduction	0.72	0.34–0.89	0.72	0.32–0.90	0.69	0.28–0.88	0.71	0.30–0.90

*Reliability indices classified as good (ICC, 0.75 – 0.90) are shown in bold type. /s, per second; CI, confidence interval; ICC, intra-class correlation coefficient*.

**Table 3:** Absolute reliability (SEM%, SDC%) of the concentric isokinetic strength assessments at 60°/s.

	**Peak torque**				**Mean peak torque**			
	**Dominant**		**Non-dominant**		**Dominant**		**Non-dominant**	
	**SEM%**	**SDC%**	**SEM%**	**SDC%**	**SEM%**	**SDC%**	**SEM%**	**SDC%**
Knee extension	15.65	**43.26**	18.17	50.21	15.71	**43.40**	19.60	54.17
Knee flexion	18.63	51.47	12.64	**34.94**	18.02	**49.81**	21.61	59.71
Hip extension	31.57	87.26	25.38	70.15	32.89	90.91	46.22	127.73
Hip flexion	23.85	65.90	22.56	62.35	21.69	59.96	22.57	62.37
Hip abduction	18.34	50.68	17.69	**48.88**	17.06	**47.16**	19.95	55.14
Hip adduction	29.68	82.02	27.83	76.93	14.94	**41.28**	23.92	66.12

*Acceptable values (≤ 50.00%) are shown in bold type. /s, per second; SDC%, smallest detectable change; SEM%, standard error of measurement*.

**Table 4:** Absolute reliability (SEM%, SDC%) of the concentric isokinetic strength assessments at 90°/s.

	**Peak torque**				**Mean peak torque**			
	**Dominant**		**Non-dominant**		**Dominant**		**Non-dominant**	
	**SEM%**	**SDC%**	**SEM%**	**SDC%**	**SEM%**	**SDC%**	**SEM%**	**SDC%**
Knee extension	22.84	63.11	17.10	**47.26**	23.80	65.78	18.55	51.25
Knee flexion	45.91	126.89	22.15	61.21	20.61	56.96	24.01	66.38
Hip extension	30.09	83.16	21.83	60.35	29.71	82.10	23.87	65.98
Hip flexion	25.93	71.67	24.15	66.75	26.84	74.19	24.25	67.01
Hip abduction	19.02	52.57	11.16	**30.85**	18.06	**49.91**	11.49	**31.77**
Hip adduction	20.27	56.03	18.07	**49.93**	22.15	61.22	18.62	51.46

*Acceptable values (≤ 50.00%) are shown in bold type. /s, per second; SDC%, smallest detectable change; SEM%, standard error of measurement*.

#### Relative Reliability

Assessments of knee flexion and knee extension at 60°/s showed good relative reliability for the PT values, with ICCs of 0.84 and 0.87 (dominant leg) and 0.89 and 0.81 (non-dominant leg), respectively. MPT results at 60°/s showed good relative reliability for knee flexion and knee extension, both for dominant (ICCs of 0.85 and 0.88, respectively), and non-dominant side (ICCs of 0.88 and 0.79, respectively). Assessments at 90°/s showed good relative reliability for knee flexion of the dominant leg for PT and MPT values (ICC 0.75 and 0.80, respectively). Further, the PT and MPT for knee extension of the non-dominant leg (ICC 0.83 and 0.80, respectively), showed good reproducibility. Knee extension of the dominant leg and knee flexion of the non-dominant leg showed moderate relative reliability (PT and MPT values) of 0.58–0.67.

Isokinetic assessment of hip extension at 60°/s for the non-dominant leg (PT and MPT value) showed good relative reliability (ICC 0.75 and 0.76, respectively), while poor to moderate results were recorded for all other assessments of both hip extension and flexion (0.50–0.73). Assessments of PT values of hip abduction showed good relative reliability for hip abduction for both sides and velocity conditions (0.77–0.88). The same results were observed for MPT values except for hip abduction at 60°/s of the non-dominant leg. Hip adduction showed overall moderate reproducibility (0.60–0.75).

#### Absolute Reliability

The SEM% values for knee flexion and extension were highly variable, with the lowest for knee flexion of the non-dominant leg at 60°/s (PT; 12.6%) and the highest for knee flexion of the dominant leg at 90°/s (PT; 45.9%). This variability is also reflected in the relatively large standard deviations. With exception of the knee flexion of the dominant leg at 90°/s (PT), the SDC% values ranged from 34.9% for knee flexion of the non-dominant leg at 60°/s (PT) to 66.4% for knee flexion of the non-dominant leg at 90°/s (MPT).

The SEM% values for the hip assessments showed the same patterns as the relative reliability results. For hip flexion and extension SDC% values were above 50%. The SDC% were ≤ 50% for hip abduction except for PT value of the dominant leg at 60°/s and 90°/s (50.7% and 52.6%, respectively) and the MPT value of the non-dominant leg at 60°/s (55.1%). For hip adduction, only the PT value of the non-dominant leg at 90°/s (49.9%) and MPT value of the dominant leg at 60°/s (41.3%) were ≤ 50%.

### Associations

Table 5 shows the associations between the MPT values of the concentric isokinetic strength assessments and the functional strength tests.

**Table 5:** Associations between concentric isokinetic strength assessments (mean peak torque) and the functional strength tests.

	**Knee extension**				**Hip extension**				**Hip abduction**			
	**60**		**90**		**60**		**90**		**60**		**90**	
	**D**	**ND**	**D**	**ND**	**D**	**ND**	**D**	**ND**	**D**	**ND**	**D**	**ND**
STS	**0.51**	0.12	0.42	0.19	0.22	–0.13	0.05	–0.39	**0.52**	0.25	**0.53**	0.25
LSU_D	0.39	/	0.03	/	0.14	/	0.12	/	0.33	/	0.30	/
LSU_ND	/	0.14	/	0.18	/	0.13	/	0.15	/	0.32	/	0.28

*Significant values are shown in bold type. D, dominant; LSU, lateral-step-up; ND, non dominant; STS, sit-to-stand*.

Moderate significant positive correlations were found between the concentric isokinetic strength assessment of the knee extensors at 60°/s, hip abductors at 60°/s and 90°/s (dominant leg) and the STS (*r* = 0.51–0.53). All other correlations were weak to very weak.

## Discussion

The main purpose of the present study was to analyze the relative and absolute reliability of concentric isokinetic strength assessments of the knee and hip in healthy children. This study tended to highlight the importance of reliable strength assessments of the lower limbs in healthy children. Both relative and absolute reliability were analyzed to emphasize the usability of concentric isokinetic strength assessments in clinical practice. Besides, the relationship between concentric isokinetic strength assessments and functional strength tests was analyzed.

### Torques

Torque values for knee and hip found in this study were rather similar to results found in previous literature (Burnett et al., 1990; Fagher et al., 2016). However, in the present study, the standard deviations appeared to be larger, which might be partially explained by the larger age range compared to other studies.

### Intra-Tester Reliability

In comparison with the results of Fagher et al. (2016), reporting an ICC of 0.62 for knee flexion and 0.81 for knee extension at 60°/s using the Biodex System 4, better results for knee flexion and for knee extension were found in current study. The better results might be partially explained by the larger variation in age of the participants (10 ± 1.6 y) compared to Fagher et al. (2016) (8.8 ± 0.5 y). Another parameter that could have influenced the results is the use of gravity correction in the study of Fagher et al. (2016). Knowing that limb weight is difficult to assess in children, variations in those values might have influenced their results resulting in lower reliability indices (ICC).

Burnett et al. (1990) reported good relative reliability for PT values at 90°/s for hip flexion (ICC 0.75) and extension (ICC 0.84), and reported poor to moderate relative reliability results for hip adduction (0.49) and abduction (0.59) using the Cybex II dynamometer. The current study showed better results for hip adduction and abduction which might be related to a better standardization of the testing procedure with a positioning of the children (with backrest) that allowed less compensatory movements.

The clinical value of a measurement device can be derived from the magnitude of the absolute reliability, as reflected by the SEM% and the SDC%. Hereby, changes over time and thus effectiveness of interventions can be determined. In current study highly variable SEM% values were found and those values tended to be smaller at 60°/s. This tendency for better results at lower velocity conditions was also reported by Fagher et al. (2016) and is in line with the results for relative reliability. It is known children have reduced ability to recruit a greater percentage of motor units, compared to adults (Amstrong, 2017). An immature neuromuscular activation pattern in children could explain why they have more difficulties being consistent at higher velocities (De Ste Croix et al., 2003; Fagher et al., 2016). The SDC% reflects the threshold to define a real change in a single subject. Youth strength training interventions often result in benefits of up to 50% strength gain (Dahab and McCambridge, 2009). For knee flexion and extension, and hip abduction SDC% values were around 50%. Fagher et al. (2016) reported better results for knee flexion and extension of the dominant leg at 60°/s (30.9% and 36.5%, respectively).

Regarding the hip, Burnett et al. (1990) did not report absolute reliability results, so no comparison can be made.

Overall, the better results for knee flexion and extension and hip abduction might be influenced by the fact that a more standardized testing procedure could be adopted, e.g., the backrest in side-lying position when testing hip abduction, allowing more isolated movements. In opposition, compensatory movements of the pelvis and of the shank segment during hip flexion and hip extension might have influenced the moderate reliability results. Also, knee flexion and extension in a seated position are movements that children are familiar with in daily life, easier to understand and to execute in a selective way, which may also have contributed to these good results. Hip adduction and hip extension, respectively in side-lying and supine position, might be more difficult to understand and perform.

### Associations

In the current study, few associations between concentric isokinetic strength and the functional strength tests were found. Only knee extension and hip abduction at 60°/s and hip abduction at 90°/s of the dominant side correlated significantly with the STS test. Those results suggest that the strength of the dominant leg would have a greater influence on the performance of the STS compared to the non-dominant leg. The STS also tended to show more associations to strength generated at a lower velocity (60°/s). The assessments at 60°/s tend to show better reliability results and it is easier to generate maximum strength at lower velocities, which might explain the better associations of the functional strength tests with torques generated at a lower angular velocity. No associations were found with isokinetic strength of the hip extensors. This could be due to the weaker reliability of the isokinetic assessment of the hip extensors in the current study. Concentric activity of the hip extensors might also be less important during the STS movement compared to the activity of the knee extensors and hip abductors. Regarding the LSU, no associations were found with the isokinetic assessments. This might be due to some confounding factors that are more difficult to objectify during the LSU, such as the amount of support children took on the wall in front of them, the way they touched the ground with the non-tested leg or the amount of lateral dipping of the hip. Besides, the range of motion (ROM) of the knee and the hip during the LSU is smaller compared to the STS, which could make it more difficult to associate it with muscular strength. Future research is needed to provide more solid conclusions. In general, the results of the associations in the current study are not very consistent and associations are scarce. These findings are in accordance with the results reported by Duncan et al. (2018), suggesting that concentric isokinetic muscle strength might not fully represent the muscular and motor performance demands of functional strength tests, which also involve components such as balance and coordination and are more likely to be considered as endurance tests. Dynamometers test the performance of one joint at a time in an open kinetic chain, whereas functional strength tests may give a more accurate view of overall limb function in closed kinetic chain activities. This is in line with the concept of specificity of training, in which the carry-over of gains made in strength in the open chain into the closed chain function is questioned (Palmitier et al., 1991; Worrell et al., 1993). Also in adults, the relationship between isokinetic strength and functional tests has been studied previously. Nevertheless, results are inconclusive varying from small to large correlations (Vassis et al., 2020). Differences in associations might be due to the variety in population (different pathological conditions e.g. total knee replacement or patellofemoral problems) and methodologies (selected functional strength tests, angular velocities, isokinetic outcomes) (Wilk et al., 1994; Yoshida et al., 2008; Guney et al., 2016). A study conducted in healthy adults evaluated the effects of a lateral-step-up exercise protocol on isokinetic peak torque of the knee extensors (Worrell et al., 1993). They concluded that an isokinetic dynamometer was unable to detect the strength gains that resulted from increases in lower extremity performance. Isokinetic strength assessments and functional strength seem to represent different aspects of the ability to perform functional tasks and might be seen as complementary.

### Limitations and Recommendations for Future Research

The present study has some limitations that could be addressed in further research. Firstly, the sample size of this study was rather small. The minimum required number of participants to obtain a correct estimate of the reliability (at least 15) was recruited (Fleiss, 2011). To express usable SDC%, a greater sample of 30–50 participants is recommended (Hopkins, 2000). Secondly, the age range of the participants included in this study was large (6 to 12 y). Differences in performance of strength assessments could be expected in younger children compared to older children. A study conducted in young boys (6 to 8 y; *n* = 12) using a Lido Active dynamometer reported good relative reliability for knee flexion and knee extension (ICC, 0.85 and 0.95, respectively) at 100°/s (Merlini et al., 1995). Nevertheless, future studies should be able to define the reliability in different age groups and compare the outcomes, using the same testing protocol. Although Tsiros et al. (2011) showed that there is no need for a separate familiarization session for concentric isokinetic strength testing of the knee in children, this might not be the case for the assessments of the hip joint. Future research should investigate the impact of a separate familiarization session for isokinetic strength assessment of the hip. To enhance standardization and avoid compensatory movements during hip abduction and adduction, a backrest was used. In order to improve reliability of concentric isokinetic strength assessments of the hip flexors and extensors, future research could implement the use of a brace to avoid movements of the shank (Meyer et al., 2013). Another limitation is the fact that only PT and MPT values were taken for analyses. Isokinetic dynamometers offer a wide range of data that can be examined and give information about the quality of performance. Other variables (e.g. total work, average power, time to peak torque) could be taken for analysis in future research. To study associations with the timed functional strength tests, endurance protocols could be used. At last, given the knowledge that eccentric muscle strength plays an important role in functional activities, it could be valuable to study associations of eccentric strength assessments with functional tests. Nevertheless, more research is needed to give an insight on the reliability of eccentric isokinetic strength assessments of the knee and hip in children.

### Conclusion and Clinical Implications

Concentric isokinetic strength assessments with the Biodex System 4 device in healthy children show moderate to good reproducibility with good relative reliability for knee flexion, knee extension and hip abduction and moderate to good relative reliability for hip extension, hip flexion, and hip adduction. To measure changes in time, knee flexion, knee extension, and hip abduction had acceptable absolute reliability. Both relative and absolute reliability at 60°/s tended to show slightly better results compared to reliability at 90°/s. Since no differences were found between outcome values of different velocities and between the dominant and non-dominant side, as well as no substantial differences in reliability indices, we would recommend to limit the assessments to one velocity (60°/s) and one side (dominant side) in order to be able to optimize the testing protocol. Because MPT may be considered as a better estimate for overall function compared to PT, we would suggest to use MPT values.

Few correlations were found between concentric isokinetic strength and the functional strength tests which means other variables such as muscle endurance, balance and coordination are to be taken into consideration if functional performance is targeted. Isokinetic strength assessments and functional strength seem to represent different aspects of the ability to perform functional tasks and might be seen as complementary.

## Data Availability Statement

The raw data supporting the conclusions of this article will be made available by the authors, without undue reservation.

## Ethics Statement

The studies involving human participants were reviewed and approved by Ethics Committee of Ghent University Hospital. Written informed consent to participate in this study was provided by the participants' legal guardian/next of kin. Written informed consent was obtained from the minor(s)' legal guardian/next of kin for the publication of any potentially identifiable images or data included in this article.

## Author Contributions

VvT: study conception and design, data collection, analysis and interpretation of the data, and drafting the manuscript. IA-G, PC: analysis and interpretation of the data, drafting the manuscript. BH, LH, HF, and KD: interpretation of the data and drafting the manuscript. CV: study conception and design, analysis and interpretation of the data, and drafting the manuscript. All authors contributed to the article and approved the submitted version.

## Conflict of Interest

The authors declare that the research was conducted in the absence of any commercial or financial relationships that could be construed as a potential conflict of interest.

## Publisher's Note

All claims expressed in this article are solely those of the authors and do not necessarily represent those of their affiliated organizations, or those of the publisher, the editors and the reviewers. Any product that may be evaluated in this article, or claim that may be made by its manufacturer, is not guaranteed or endorsed by the publisher.

## Abbreviations

Cm: centimeters
ICC: intra class correlations
Kg: kilogram
LSU: lateral-step-up
MPT: mean peak torque
Nm: Newton meter
PT: peak torque
r: Pearson correlation coefficient
s: second
SEM: standard error of measurement
SDC: smallest detectable change
STS: sit-to-stand
y: years.
