# Supplementary material for: Reliability of Isokinetic Strength Assessments of Knee and Hip Using the Biodex System 4 Dynamometer and Associations With Functional Strength in Healthy Children
Source: Front Sports Act Living. 2022 Feb 24;4:817216. doi: 10.3389/fspor.2022.817216 (PMC8907626; doi:10.3389/fspor.2022.817216)
Supplement: Supplementary file 1 [file Table_1.DOCX]

APPENDIX – Table A1

Peak Torques and Mean Peak Torques of the Knee

| **Assessments** | **N** | **Mean** | **SD** | **Assessments** | **N** | **Mean** | **SD** |
| --- | --- | --- | --- | --- | --- | --- | --- |
| **Knee Extension (60°- Dominant)** | | | | **Knee Flexion (60°- Dominant)** | | | |
| 1st (max) | 13 | 51.95 | 23.36 | 1st (max) | 11 | 28.87 | 14.10 |
| 2nd (max) | 13 | 52.05 | 22.88 | 2nd (max) | 12 | 29.97 | 12.60 |
| 1st (mean) | 13 | 48.68 | 22.98 | 1st (mean) | 11 | 27.34 | 13.57 |
| 2nd (mean) | 13 | 49.24 | 21.22 | 2nd (mean) | 12 | 27.92 | 11.78 |
| **Knee Extension (90°- Dominant)** | | | | **Knee Flexion (90°- Dominant)** | | | |
| 1st (max) | 15 | 51.89 | 19.82 | 1st (max) | 17 | 27.23 | 14.05 |
| 2nd (max) | 15 | 51.9 | 17.58 | 2nd (max) | 17 | 27.82 | 10.23 |
| 1st (mean) | 15 | 49.36 | 19.92 | 1st (mean) | 17 | 25.26 | 13.36 |
| 2nd (mean) | 15 | 47,95 | 16.21 | 2nd (mean) | 17 | 25.89 | 10.11 |
| **Knee Extension (60°- Non dominant)** | | | | **Knee Flexion (60°- Non dominant)** | | | |
| 1st (max) | 15 | 48.26 | 22.74 | 1st (max) | 14 | 30.24 | 12.57 |
| 2nd (max) | 14 | 48.83 | 17.37 | 2nd (max) | 14 | 29.73 | 10.32 |
| 1st (mean) | 15 | 45.2 | 22.26 | 1st (mean) | 14 | 28.03 | 11.91 |
| 2nd (mean) | 14 | 44.39 | 15.12 | 2nd (mean) | 14 | 27.06 | 9.39 |
| **Knee Extension (90°- Non dominant)** | | | | **Knee Flexion (90°- Non dominant)** | | | |
| 1st (max) | 18 | 47.15 | 20.12 | 1st (max) | 14 | 28.12 | 11.69 |
| 2nd (max) | 18 | 44.3 | 17.85 | 2nd (max) | 14 | 25.71 | 8.85 |
| 1st (mean) | 18 | 43.81 | 18.59 | 1st (mean) | 14 | 25.69 | 10.94 |
| 2nd (mean) | 18 | 41.6 | 16.89 | 2nd (mean) | 14 | 23.78 | 8.28 |
| max: Peak Torque; mean: Mean Peak Torque; N: number of participants; S: Standard Deviation; 1st: first; 2nd: second | | | | | | | |

APPENDIX - Table A2

Peak Torques and Mean Peak Torques of the Hip Flexion and Extension

| **Assessments** | **N** | **Mean** | **SD** | **Assessments** | **N** | **Mean** | **SD** |
| --- | --- | --- | --- | --- | --- | --- | --- |
| **Hip Extension (60°- Dominant)** | | | | **Hip Flexion (60°-Dominant)** | | | |
| 1st (max) | 17 | 30.62 | 17.18 | 1st (max) | 14 | 39.51 | 16.01 |
| 2nd (max) | 17 | 31.15 | 12.06 | 2nd (max) | 14 | 35.05 | 12.21 |
| 1st (mean) | 17 | 28.76 | 16.52 | 1st (mean) | 14 | 37.12 | 15.89 |
| 2nd (mean) | 17 | 29.01 | 11.13 | 2nd (mean) | 14 | 32.38 | 11.47 |
| **Hip Extension (90°- Dominant)** | | | | **Hip Flexion (90°- Dominant)** | | | |
| 1st (max) | 17 | 30.66 | 16.67 | 1st (max) | 15 | 36.87 | 13.09 |
| 2nd (max) | 16 | 31.88 | 12.15 | 2nd (max) | 15 | 31.78 | 12.88 |
| 1st (mean) | 16 | 30.34 | 14.06 | 1st (mean) | 15 | 34.13 | 11.56 |
| 2nd (mean) | 16 | 29.24 | 11.20 | 2nd (mean) | 15 | 29.27 | 11.90 |
| **Hip Extension (60°- Non dominant)** | | | | **Hip Flexion (60°- Non dominant)** | | | |
| 1st (max) | 16 | 34.28 | 17.51 | 1st (max) | 18 | 40.75 | 17.35 |
| 2nd (max) | 16 | 30.73 | 15.27 | 2nd (max) | 18 | 36.11 | 13.19 |
| 1st (mean) | 16 | 31.90 | 16.25 | 1st (mean) | 18 | 37.82 | 17.14 |
| 2nd (mean) | 16 | 30.54 | 12.35 | 2nd (mean) | 18 | 31.93 | 11.63 |
| **Hip Extension (90°- Non dominant)** | | | | **Hip Flexion (90°- Non dominant)** | | | |
| 1st (max) | 14 | 30.96 | 14.34 | 1st (max) | 19 | 37.86 | 16.31 |
| 2nd (max) | 14 | 31.68 | 9.00 | 2nd (max) | 19 | 31.29 | 11.86 |
| 1st (mean) | 14 | 28.72 | 13.98 | 1st (mean) | 19 | 34.86 | 15.54 |
| 2nd (mean) | 14 | 29.42 | 9.01 | 2nd (mean) | 19 | 29.53 | 11.34 |
| max: Peak Torque; mean: Mean Peak Torque; N: number of participants; S: Standard Deviation; 1st: first; 2nd: second | | | | | | | |

APPENDIX - Table A3

Peak Torques and Mean Peak Torques of the Hip Abduction and Adduction

| Assessments | N | Mean | SD | Assessments | N | Mean | SD |
| --- | --- | --- | --- | --- | --- | --- | --- |
| **Hip Abduction (60°- Dominant)** | | | | **Hip Adduction (60°- Dominant)** | | | |
| 1st (max) | 16 | 29.06 | 11.80 | 1st (max) | 14 | 32.13 | 9.14 |
| 2nd (max) | 16 | 33.11 | 13.75 | 2nd (max) | 14 | 32.30 | 9.24 |
| 1st (mean) | 16 | 27.12 | 11.55 | 1st (mean) | 14 | 29.65 | 8.34 |
| 2nd (mean) | 16 | 30.07 | 12.14 | 2nd (mean) | 14 | 30.07 | 9.02 |
| **Hip Abduction (90°- Dominant)** | | | | **Hip Adduction (90°- Dominant)** | | | |
| 1st (max) | 14 | 29.32 | 14.27 | 1st (max) | 15 | 28.65 | 11.49 |
| 2nd (max) | 14 | 31.47 | 14.88 | 2nd (max) | 15 | 29.76 | 10.90 |
| 1st (mean) | 14 | 27.21 | 13.41 | 1st (mean) | 15 | 27 | 11.36 |
| 2nd (mean) | 14 | 29.11 | 13.49 | 2nd (mean) | 15 | 27.63 | 10.51 |
| **Hip Abduction (60°- Non dominant)** | | | | **Hip Adduction (60°- Non dominant)** | | | |
| 1st (max) | 15 | 30.39 | 10.91 | 1st (max) | 14 | 28.98 | 8.94 |
| 2nd (max) | 15 | 32.6 | 12.18 | 2nd (max) | 14 | 25.63 | 14.30 |
| 1st (mean) | 15 | 27.45 | 9.30 | 1st (mean) | 14 | 27.56 | 8.52 |
| 2nd (mean) | 15 | 29.69 | 11.73 | 2nd (mean) | 14 | 24.1 | 12.14 |
| **Hip Abduction (90°- Non dominant)** | | | | **Hip Adduction (90°- Non dominant)** | | | |
| 1st (max) | 14 | 30.86 | 9.54 | *1st (max)* | 13 | 30.30 | 11.55 |
| 2nd (max) | 15 | 30.42 | 9.99 | *2nd (max)* | 13 | 28.05 | 7.85 |
| 1st (mean) | 14 | 28.06 | 8.92 | *1st (mean)* | 13 | 28.08 | 10.71 |
| 2nd (mean) | 14 | 28.15 | 10.33 | *2nd (mean)* | 13 | 25.95 | 7.52 |
| max: Peak Torque; mean: Mean Peak Torque; N: number of participants; S: Standard Deviation; 1st: first; 2nd: second | | | | | | | |
